# An improved DNA method to unambiguously detect small hive beetle Aethina tumida, an invasive pest of honeybee colonies

**DOI:** 10.1002/ps.5141

**Published:** 2018-09-15

**Authors:** Paolo Silacci, Claudine Biolley, Corinne Jud, Jean‐Daniel Charrière, Benjamin Dainat

**Affiliations:** ^1^ Agroscope Swiss Bee Research Centre, Bern Switzerland

**Keywords:** Aethina tumida, Apis mellifera, diagnostics, multiplex PCR

## Abstract

The scavenger and invasive species Aethina tumida threatening the honey bee has been recently introduced in Europe. We present a new, reliable and rapid multiplex real‐time PCR for efficient diagnostics enabling surveillance programs. © 2018 The Authors. *Pest Management Science* published by John Wiley & Sons Ltd on behalf of Society of Chemical Industry.

1

**Photo 1:**
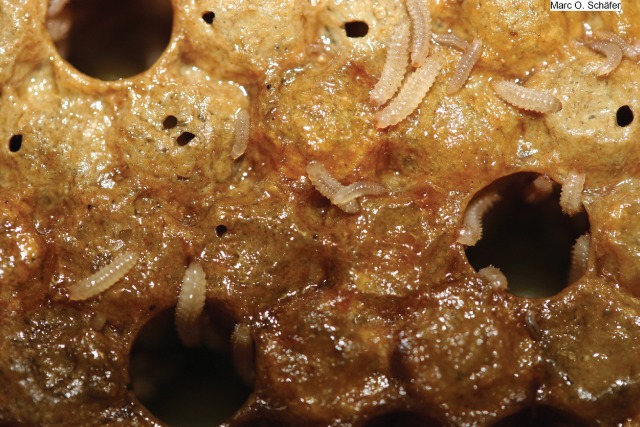
Small hive beetle larvae on brood comb.

The small hive beetle (SHB) *Aethina tumida* Murray *(Coleoptera: Nitidulidae*) is a scavenger native to sub‐Saharan Africa and is a pest of honey bees without provoking significant damage within its endemic range.^1^, ^2^ Since the first report in 1996 out of its native range^3^ in North Carolina, USA, the beetle became an invasive species in Australia, and in central and North America. It was introduced in Europe in 2004 in Portugal where an eradication program was effective and then in Italy in 2014 where infestation is still ongoing^4^ probably originating from an African population.^5^ Its life cycle is intimately linked to the honey bee where it mates and reproduces inside the colony and where the larvae feed on beebread, honey and brood causing destruction.^1^, ^6^, ^7^ Besides honey bees, it can also affect bumble bees and stingless bees (see review in reference^2^). The economic damage to the beekeeping industry can therefore be substantial thereby explaining why the SHB is a statutory notifiable pest in the European Union (EU). After its introduction in Italy, the EU authorities (Commission Implementing Decision 2014/909/EU of 12 December 2014) established new protective measures to prevent SHB spread including the goal to eradicate it if possible. It is consequently of the utmost importance to have an easy, reliable and cheap technique for diagnostics. However, the eggs and larvae stage are extremely difficult to identify with conventional taxonomic techniques bearing a too high risk of incorrect results. A previous PCR assay is based on work published in 2007,^8^ where an amplification system targeting the cytochrome oxidase subunit I (COI) gene was proposed. However, the sequence of the reverse primer contains an important internal mismatch of three nucleotides with all currently published *A. tumida* COI sequences, rendering its application tedious and susceptible to false negative diagnostic. In this study, we propose a new SHB diagnostics using a multiplex PCR approach targeting COI gene and a common region of 18S ribosomal gene as internal control.

These results further confirm the validity of the proposed multiplex PCR system for rapid, reliable and specific diagnostics of the small hive beetle (SHB) that may be found in the hive and thus facilitate for example early detection programs.

The DNA extraction method was chosen according to a previous study comparing different extraction procedures of genomic DNA from ticks providing material allowing maximal recovery and good quality for a consistent amplification.^9^ Briefly, insects stored in ethanol 70% (*v*/*v*) were air‐dried prior to dissection into four quarters and then DNA was extracted using GeneJet Genomic Kit (Thermo Fisher, Waltham, MA, USA) following the manufacturer's instructions and eluted into a final volume of 100 μL. Then, 4 μL (0.8–10 ng) of insect genomic DNA were used for amplification. PCR was performed in a 20 μL reaction volume containing 1x KAPA PROBE Fast Universal Master Mix (Sigma, St Louis, MO, USA), 200 nmoL/L of COI and 18S primers, 400 nmoL/L of COI probe and 50 nmoL/L of 18S probe. The amplification profile included an activation step of 5 min at 95 °C followed by 60 cycles of a two‐step amplification (5 s at 95 °C; 20 s at 62 °C), using an Eco™ real time PCR device (PCR^max^). Primers (Table 1) were designed with Primer‐Blast^10^ thereby checking specificity in nucleotide Databank to any known sequences and probes with Primer3 v. 0.4.0.^11^ Both primers and probes were synthetized by Microsynth (Microsynth, Balgach, Switzerland). Amplification results were analyzed with Eco Study v.5.0 (PCR^max^). Size and sequence of the COI amplicon were further verified on a DNA extracted from a beetle individual isolated in Italy (data not shown).

**Table 1:** Sequences of primers and probes targeting COI and 18S genes. Amplicon lengths were of 396 bp for COI and 80 bp for 18S

	COI gene	18S gene
Forward primers	5′‐CGACCCTCAGGCATAACCTT‐3′	5′‐AATCAGCGTGTCTTCCCTGG‐3′
Reverse primers	5′‐AGGCTCGAGTATCAACGTCTA‐3′	5′‐CAATTGCAAGCCCCAATCCC‐3′
Probes	5′‐**HEX**‐GGAAGCCTTTGGAACTTTAGG‐**BHQ‐3**′	5′‐**FAM**‐GTAACCCGCTGAACCTCCTT‐**BHQ**‐3′

After optimization of the concentration of the two probes, to further confirm the specificity of the primers the multiplex assay was tested on a total of 49 DNAs extracted from different insects which can be found in the vicinity of colony hives or are common in European fields. The DNA test set included 12 *A. tumida* individuals of different geographical origins and relatives from the Nitidulidae family. All the DNA extracted from *A. tumida* were positive for both the internal control 18S and COI gene amplification. Interestingly, threshold cycles (Cq) for the two systems never diverged for more than 6.4 cycles (Table 2). All the other 37 insect samples analyzed were positive for 18S, with Cq ≤ 42.1, and negative for COI (Table 2).

**Table 2:** Results of multiplex Aethina tumida PCR system application to 49 different insect DNA

Individual	Species with GBOLD accession number where relevant	Origin	Development stage	Atum Cq	18S Cq
**1**	*Aethina tumida*	Italy	Adult	28.7	28.6
**2**	*Aethina tumida*	Italy	Larvae	30.9	26.4
**3**	*Aethina tumida*	Italy	Larvae	32.5	26.1
**4**	*Aethina tumida*	Italy (Calabria)	Adult	21	22.8
**5**	*Aethina tumida*	Italy (Calabria)	Adult	20.2	22.1
**6**	*Aethina tumida*	United Kingdom (breeding from an US strain)	Larvae	35.2	28.2
**7**	*Aethina tumida*	United Kingdom (breeding from an US strain)	Adult	30.3	26.2
**8**	*Aethina tumida*	Mexico	Adult	18.8	21.4
**9**	*Aethina tumida*	South Africa	Adult	27.9	34.1
**10**	*Aethina tumida*	South Africa	Adult	28	33.4
**11**	*Aethina tumida*	South Africa	Larvae	28	31.5
**12**	*Aethina tumida*	South Africa	Larvae	22.4	30
**13**	*Harmonia axyridis*	Switzerland	Adult	ND	16.1
**14**	*Harmonia axyridis*	Switzerland	Adult	ND	23.5
**15**	*Muscidae*	Switzerland	Adult	ND	42.1
**16**	*Forficula auricularia*	Switzerland	Adult	ND	38.5
**17**	*Leptoglossus occidentalis*	Switzerland	Adult	ND	37.1
**18**	*Galleria mellonella*	Switzerland	Larvae	ND	21.4
**19**	*Galleria mellonella*	Switzerland	Larvae	ND	22.8
**20**	*Lepidoptera*	Switzerland	Larvae	ND	32.7
**21**	*Varroa destructor*	Switzerland	Adult	ND	31
**22**	*Meligethes viridescens*	Switzerland	Adult	ND	35
**23**	*Cychramus luteus (Nitidulidae)* ZFMK‐TIS‐2504554	Germany	Adult	ND	34.9
**24**	*Cychramus luteus (Nitidulidae)* ZFMK‐TIS‐2503863	Germany	Adult	ND	33.3
**25**	*Cychramus luteus (Nitidulidae)* ZFMK‐TIS‐2506747	Italy	Adult	ND	34.7
**26**	*Epuraea aestiva (Nitidulidae)* ZFMK‐TIS‐13931	Germany	Adult	ND	24.3
**27**	*Epuraea aestiva (Nitidulidae)* ZFMK‐TIS‐2504534	Germany	Adult	ND	29.4
**28**	*Epuraea aestiva (Nitidulidae)* ZFMK‐TIS‐2504535	Germany	Adult	ND	28.4
**29**	*Glischrochilus hortensis (Nitidulidae)* ZFMK‐TIS‐2522755	Germany	Adult	ND	30.6
**30**	*Glischrochilus hortensis (Nitidulidae)* ZFMK‐TIS‐11274	Germany	Adult	ND	37.5
**31**	*Glischrochilus hortensis (Nitidulidae)* ZFMK‐TIS‐11650	Germany	Adult	ND	29.5
**32**	*Glischrochilus quadriguttatus (Nitidulidae)* ZFMK‐TIS‐2515238	Germany	Adult	ND	27.9
**33**	*Glischrochilus quadriguttatus (Nitidulidae)* ZFMK‐TIS‐2511771	Germany	Adult	ND	31.4
**34**	*Glischrochilus quadriguttatus (Nitidulidae)* ZFMK‐TIS‐2521080	Germany	Adult	ND	25.4
**35**	*Glischrochilus quadriguttatus (Nitidulidae)*	Switzerland	Adult	ND	35.2
**36**	*Glischrochilus quadriguttatus (Nitidulidae)*	Switzerland	Adult	ND	34.9
**37**	*Glischrochilus quadriguttatus (Nitidulidae)*	Switzerland	Adult	ND	30.9
**38**	*Carpophilus sexpustulatus (Nitidulidae)* ZFMK‐TIS‐2521034	Germany	Adult	ND	29.1
**39**	*Carpophilus sexpustulatus (Nitidulidae)* ZFMK‐TIS‐2521033	Germany	Adult	ND	26.4
**40**	*Carpophilus sexpustulatus (Nitidulidae)* ZFMK‐TIS‐2527002	Germany	Adult	ND	29.3
**41**	*Carpophilus* sp. *(Nitidulidae)* ZFMK‐TIS‐2580556	Slovenia	Adult	ND	27
**42**	*Soronia grisea (Nitidulidae)*	Switzerland	Adult	ND	35.7
**43**	*Soronia grisea (Nitidulidae)*	Switzerland	Adult	ND	38.2
**44**	*Soronia grisea (Nitidulidae)*	Switzerland	Adult	ND	29.9
**45**	*Trichodes alvearius*	Switzerland	Adult	ND	28.6
**46**	*Hoplia philanthus (Scarabaeidae)*	Switzerland	Adult	ND	17.7
**47**	*Hoplia philanthus (Scarabaeidae)*	Switzerland	Adult	ND	18.4
**48**	*Hoplia philanthus (Scarabaeidae)*	Switzerland	Adult	ND	19.3
**49**	*Hoplia philanthus (Scarabaeidae)*	Switzerland	Adult	ND	19.9

Note: ND, non detected.

Some DNA from insects (individuals 23–34 and 38–41) were obtained from BOLD Germany, details can be found at https://doi.org/10.5883/DS-AETHINA

These observations prove the reliability of our multiplex PCR system, despite single nucleotide mismatches present in the COI reverse primer and at 5′ extremity of the COI probe with three known sequences of *A. tumida* (KT380625.1, KT380626.1, AF227647.1). These single nucleotide mismatches apparently did not influence COI amplification, nor its specificity.

These results further confirm the validity of the proposed multiplex PCR system for a rapid, reliable and specific diagnostics of SHB that may be found in the hive and thus facilitate for example early detection programs.
